# Supplementary material for: Discrimination of SARS-CoV-2 Omicron Sublineages BA.1 and BA.2 Using a High-Resolution Melting-Based Assay: a Pilot Study
Source: Microbiol Spectr. 2022 Jul 21;10(4):e01367-22. doi: 10.1128/spectrum.01367-22 (PMC9430517; doi:10.1128/spectrum.01367-22)
Supplement: Supplemental file 1 — Supplemental material. Download spectrum.01367-22-s0001.pdf, PDF file, 1.0 MB [file spectrum.01367-22-s0001.pdf]

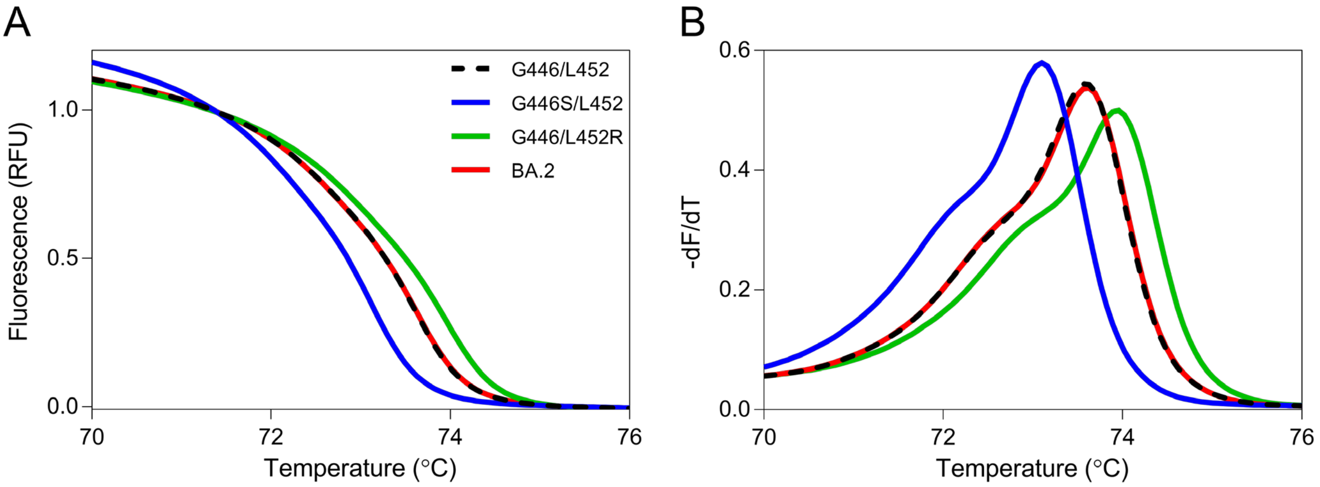

Fig. S1. Normalized melting curves and melting peaks of positive control RNAs for the R446/L452 site. Normalized melting curve plots (A) and melting peak plots (B) for the R446/L452 site were acquired using standard fragments of the G446/L452 RBD (dashed black line), G446S/L452 RBD (solid blue line), G446/L452R RBD (solid green line), and BA.2 RBD (solid red line).

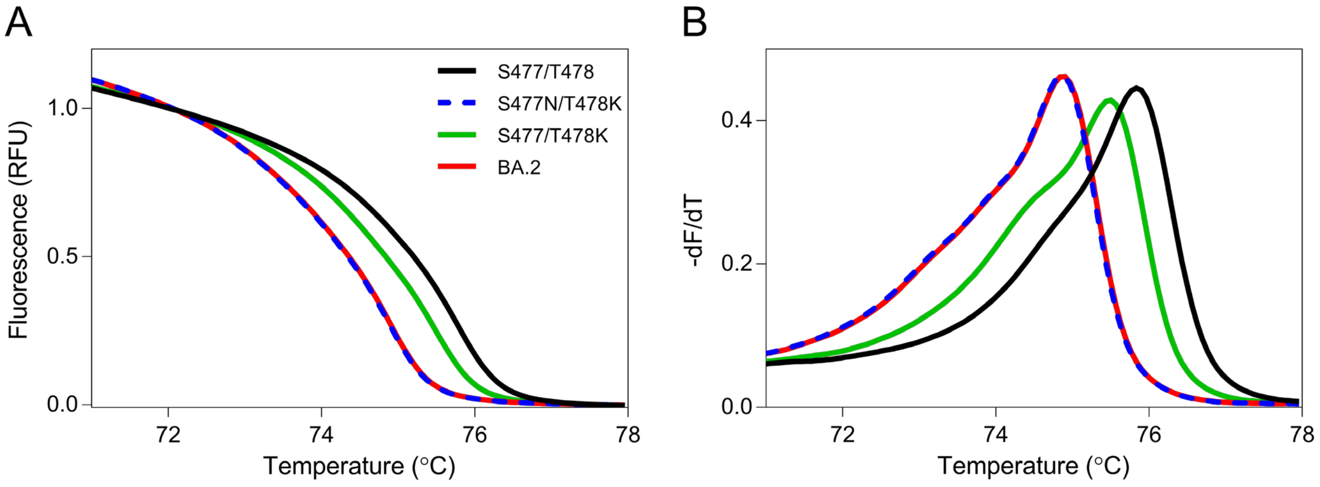

Fig. S2. Normalized melting curves and melting peaks of positive control RNAs for the S477/T478 site. Normalized melting curve plots (A) and melting peak plots (B) for the S477/T478 site were acquired using standard fragments of the S477/T478 RBD (solid black line), S477/T478 RBD (dashed blue line), S477/T478 RBD (solid green line), and BA.2 RBD (solid red line).

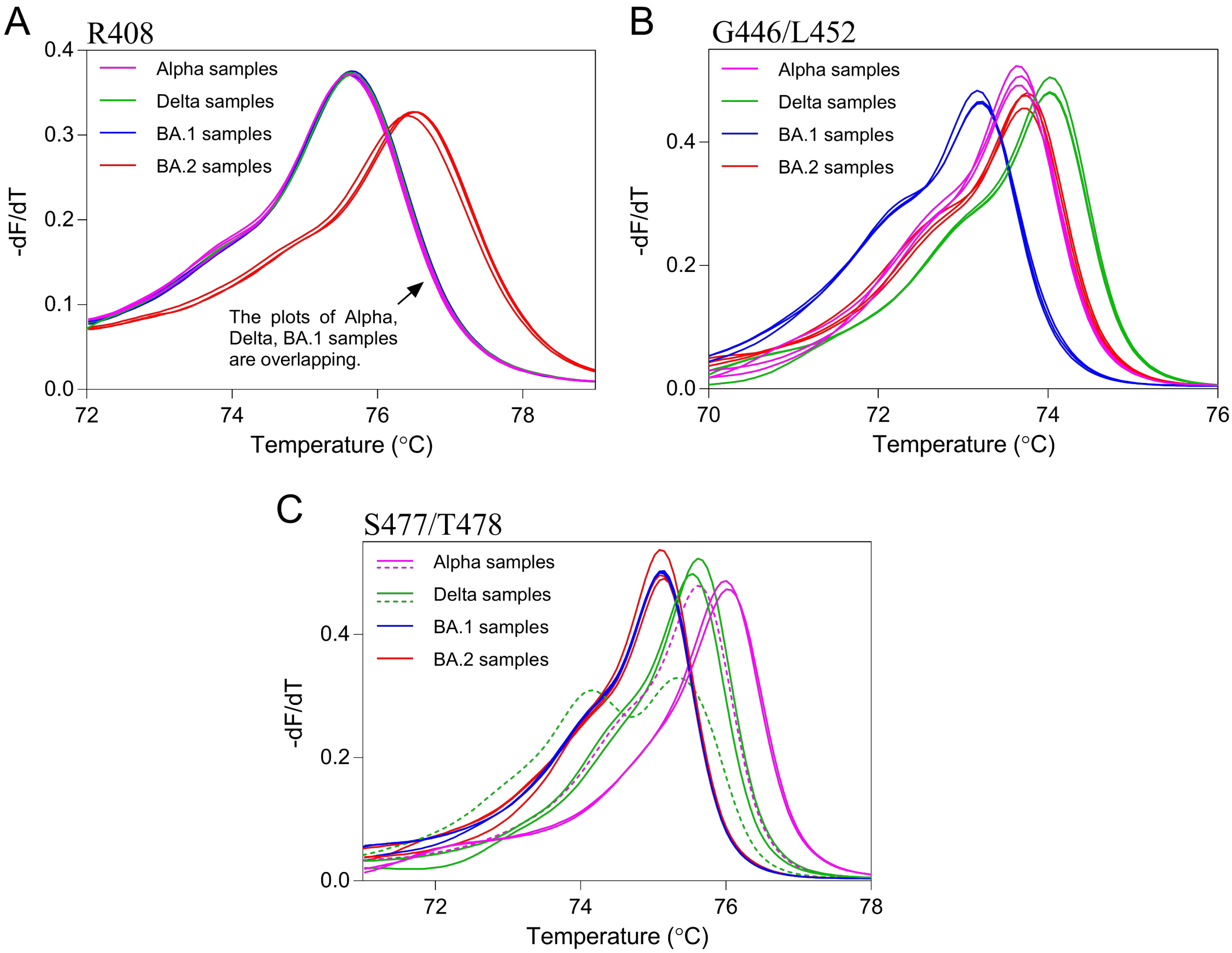

Fig. S3. Melting peaks of clinical samples for three RBD sites. Melting peak plots for the R408 (A), G446/L452 (B), and S477/T478 sites were acquired using clinical samples with three Alpha variants (pink line), three Delta variants (green line), three BA.1/BA.1.1 variants (blue line), and three BA.2 variants (red line). Solid lines indicate true positive and true negative samples. Dashed lines indicate false negative samples.
